# A Thermal Equilibrium Analysis of Line Contact Hydrodynamic Lubrication Considering the Influences of Reynolds Number, Load and Temperature

**DOI:** 10.1371/journal.pone.0134806

**Published:** 2015-08-05

**Authors:** Xiaoli Yu, Zheng Sun, Rui Huang, Yu Zhang, Yuqi Huang

**Affiliations:** Department of Energy Engineering, Zhejiang University, Hangzhou, 310027, China; Michigan State University, UNITED STATES

## Abstract

Thermal effects such as conduction, convection and viscous dissipation are important to lubrication performance, and they vary with the friction conditions. These variations have caused some inconsistencies in the conclusions of different researchers regarding the relative contributions of these thermal effects. To reveal the relationship between the contributions of the thermal effects and the friction conditions, a steady-state THD analysis model was presented. The results indicate that the contribution of each thermal effect sharply varies with the Reynolds number and temperature. Convective effect could be dominant under certain conditions. Additionally, the accuracy of some simplified methods of thermo-hydrodynamic analysis is further discussed.

## Introduction

Hydrodynamic lubrication has been used extensively to reduce friction loss between metal surfaces and the corresponding wear. In 1886, Osborne Reynolds proposed the famous Reynolds equation, which marked the beginning of theoretical hydrodynamic lubrication analysis. Gradually, some researchers [1, 2] recognized the importance of including thermal effects in hydrodynamic lubrication analysis. In recent years, to achieve the higher lubrication and cooling demands of engineering design, research on thermal effects has become meaningful and important because of its great influence on lubrication performance [3–6].

In 1962, Dowson [7] improved the previous studies and proposed the thermo-hydrodynamic (THD) theory, in which variations of temperature, viscosity and heat transfer within an oil film were considered. Through further studies, Dowson et al.[8] and some other researchers [9, 10] found that the steady-state THD condition includes three forms of thermal effects: viscous dissipation produced by the shear-rate within the oil film; conduction from or to the metal surfaces; and convection within the lubricant. When considering the unsteady-state THD conditions, the change of internal energy of the lubricant should be taken into account.

During the last few decades, numerous studies have reported on the factors that influence the thermal effects in THD lubrication. Pierre et al. [11] studied the influences of geometric parameters and operating conditions on the THD lubrication performance of journal bearings and showed that oil film thickness, friction loss and temperature distribution are affected significantly by the bearings’ diameter, load and rotational speed; and also showed that the viscous dissipation could not be neglected in THD analysis. Chauhan et al. [12] concluded that load carrying capacity, temperature and pressure distribution are highly dependent on viscosity. Wang et al. [13] derived modified Reynolds and energy equations to investigate both thermal and cavitation effects in a journal bearing using micropolar fluids as the lubricant. Chun et al. [14, 15] compared numerically the different effects of real and constant density and specific heat parameters values on high-speed journal bearing THD analysis, and concluded that the influences of real density and specific heat values on the calculation of the temperature, load and frictional loss could not be ignored. Boncompain et al. [16] and Hatakenaka et al. [17] studied the influences of reverse flow and showed that the temperature gradient across and along the oil film has a significant effect on the lubrication behavior. Harigaya et al. [18, 19] studied the effects of viscosity and temperature on piston ring-cylinder liner lubrication of a diesel engine. The results showed that the oil film thickness increases with engine speed. An unreasonable estimation of viscosity and density would lead to a calculation error that cannot be ignored. Meanwhile, the calculated film thickness varies due to the temperatures of the ring and liner surfaces.

However, because there are numerous factors that influence the thermal effects and because the physical objects studied differ greatly, the previous reports are inconsistent regarding the relative contributions of viscous dissipation, conduction and convection to hydrodynamic thermal effects. Boncompain et al. [16] concluded that most of the heat generated was removed by convection. In contrast, Chun et al. [14] held the opinion that heat convection played only a small role in determining the friction loss and load and that convection could even be ignored in some cases. Conversely, Harigaya et al. [18, 19] showed that degrees of heat transfer due to conduction and convection vary significantly with the boundary conditions and that the amounts of conduction and convection were similar, which meant that neither should be ignored in THD lubrication analysis. In reference [20–22], the mechanisms of heat transfer in pad trust bearings have been studied in detail, and the results in reference [20] show that heat dissipation distribution varies remarkably from load and speed in pad trust bearings, which might be helpful to heat transfer analysis in journal bearings. To address the above disagreements, the variations of the proportions of dissipation, conduction and convection thermal effects with changes in the influencing factors such as temperature, load and relative speed of the metal surfaces are worthy of further investigation.

In this paper, the thermal equilibrium of line contact THD lubrication is investigated by a combination of simulation and experiment. A steady-state THD analysis model of line contact lubrication has been developed to study the influences of relative speed (Reynolds number) of motion, load and the temperatures of the oil inlet and of the metal surfaces on the thermal effects, especially the proportions of each thermal effect. In parallel, a test rig corresponding to the simulation model has also been established to measure the metal surfaces’ temperatures, the viscous heating rate and the minimum oil film thickness. The experiment data verified the credibility of simulation model.

## Materials and Methods

### Simulation Model

**Fig 1:**
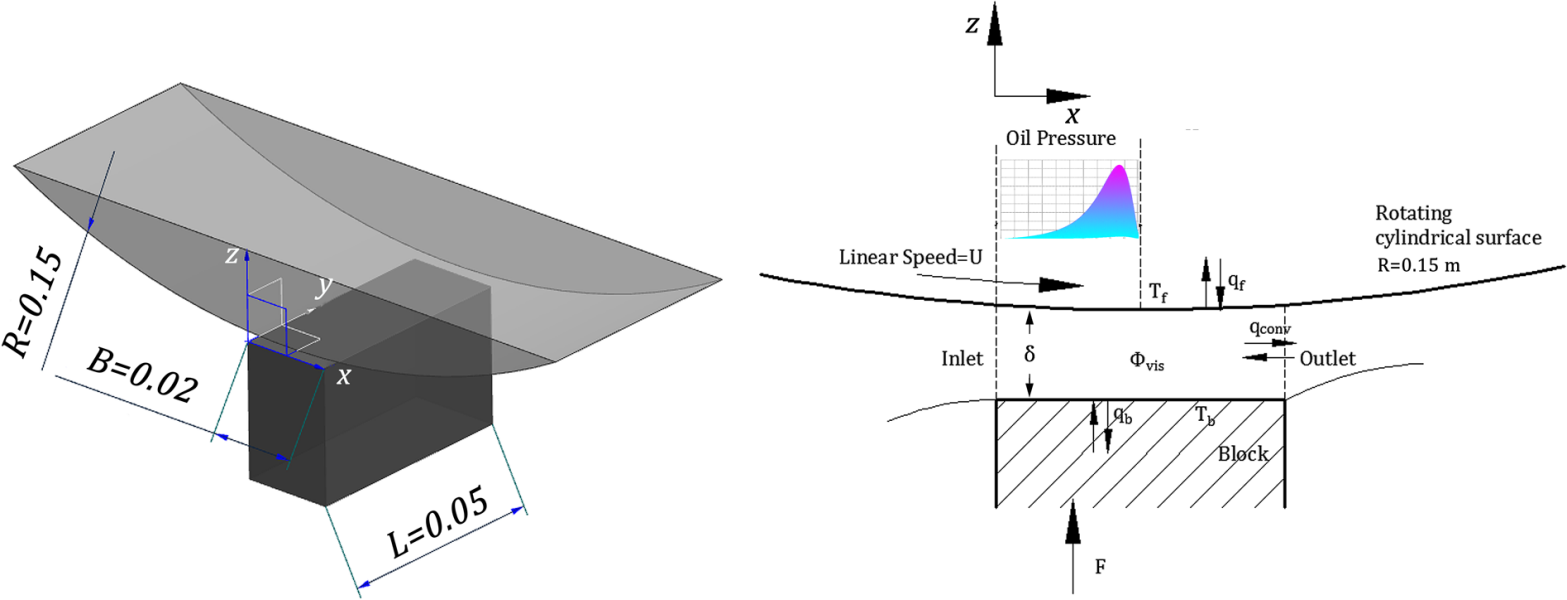
Schematic diagram of the simplified model.

As shown in Fig 1, the simplified model has been used to study the thermal equilibrium of line contact hydrodynamic lubrication. The cylindrical surface can rotate around its geometric center, and its radius is 0.15 m (>>oil film thickness). The stationary block is fixed in the *x* axis and *y* axis directions, and it is pressed by a force F onto the rotating cylindrical surface in the *z* axis direction (along the oil film thickness direction). The oil is supplied from the left side. Because of the wedge-shaped structure and the relative motion of the metal surfaces, a stable hydrodynamic lubrication film can be formed. The linear speed of the cylindrical surface varies from 4.71 to 21.60 m/s, and the inlet oil temperature varies from 60 to 90°C. The block surface temperature varies from 60 to 100°C. The applied load F varies from 220 to 380 N. The nomenclature of symbols are listed in Table 1.

**Table 1:** Nomenclature.

*p* pressure distribution in the oil film (Pa)	U linear speed of the cylindrical surface (m/s)
*η* viscosity (Pa s)	*p*_0_ atmospheric pressure (Pa)
*δ* oil film thickness (μm)	*B* stationary block width (m)
*δ*_0_ minimum oil film thickness (μm)	L stationary block length (m)
*a*_0_ correlation parameters	F applied load (N)
Re the Reynolds number	F* dimensionless load
*T*_1_ correlation parameters (°C)	u, v, w velocity component (m/s)
*T*_2_ correlation parameters (°C)	*ρ* oil density (kg/m^3^)
*T* local oil temperature (°C)	*λ* oil thermal conductivity (W/m K)
*R*_*f*_ cylindrical surface radius = 0.15 (m)	*C*_*p*_ oil specific heat (kJ/kg°C)
Φ viscous dissipation rate (W)	*T*_*b*_ stationary block surface temperature (°C)
*Q*_*b*_ conduction heat transfer rate to the block (W)	*T*_*f*_ cylindrical surface temperature (°C)
*Q*_*f*_ conduction heat transfer rate to the cylindrical surface (W)	*T*_inlet_ oil inlet temperature (°C)
*Q*_*vis*_ viscous dissipation heating rate (W)	M shaft torque under working condition (N m)
*Q*_*conv*_ convection heat transfer rate (W)	M_0_ shaft torque under idling condition (N m)
*x*,*y*,*z* spatial coordinate (m)	*u*,*v*,*w* velocity components (m/s)

#### Generalized Reynolds equation

The Reynolds equation to describe the steady-state line contact lubrication behavior may be expressed as [15, 23]

$$\frac{\partial }{\partial x}\left(\frac{\delta^{3}}{\eta }\frac{\partial p}{\partial x}\right)+\frac{\partial }{\partial y}\left(\frac{\delta^{3}}{\eta }\frac{\partial p}{\partial y}\right)=6\text{U}\frac{\partial \delta }{\partial x}$$ (1)

where *δ* and U represent the oil film thickness and linear speed of the cylindrical surface. The viscosity *η* is allowed to be temperature dependent. The exponential model[19] is assumed:

$$\eta =a_{0}exp\left(\frac{T_{1}}{T_{2}+T}\right)$$ (2)

where *a*_0_ = 7.36×10^−5^ Pa s, *T*_1_ = 1103.11°C and *T*_2_ = 113.48°C. Other data of oil 15W40 are shown in Table 2.

**Table 2:** Data of Oil 15W40.

Density	880.72Kg/m3
Specific Heat	1.985KJ/(Kg K)
Heat Conduction Coefficient	0.143W/(m K)
Viscosity (100°C)	0.0129(Pa s)
Viscosity (40°C)	0.0974(Pa s)

The oil film thickness *δ* in this paper can be defined by the expression

$$\delta =\delta_{0}+\frac{{\left(x-\frac{B}{2}\right)}^{2}}{2R_{f}}$$ (3)

where *δ*_0_, *B* and *R*_*f*_ represent the minimum oil film thickness, width of the block and radius of the flywheel.

The Half Sommerfeld Boundary Condition are used to solve the Reynolds equation:

$$p{|}_{x=0}=p{|}_{x=\frac{B}{2}}=p{|}_{y=0}=p{|}_{y=L}=p_{0}$$ (4)

*p*_0_ represents the atmospheric pressure.

#### Force balance equation

Because stable oil film lubrication has been established, the integral value of the pressure distribution should be equal to the applied load F:

$$\text{F}=\int_{x=0}^{x=B}\int_{y=0}^{y=L}p\left(x,y\right)dxdy$$ (5)

where *L* represents the length of block. This equality represents the applied load of the line contact THD lubrication.

#### CFD control equations

The mass conservation equation when density is constant:\

$$\rho \cdot \left(\frac{\partial u}{\partial x}+\frac{\partial v}{\partial y}+\frac{\partial w}{\partial z}\right)=0$$ (6)

$${u|}_{z=0}=0,\;{u|}_{z=\delta }=\text{U}$$

The velocity distribution is calculated by using the three-dimensional Navier-Stokes equations:

$$\rho \cdot \left(u\frac{\partial u}{\partial x}+v\frac{\partial u}{\partial y}+w\frac{\partial u}{\partial z}\right)=-\frac{\partial p}{\partial x}+\frac{\partial }{\partial x}\left[\eta \cdot \left(2\cdot \frac{\partial u}{\partial x}-\frac{2}{3}\cdot \left(\nabla \cdot \vec{v}\right)\right)\right]+\frac{\partial }{\partial y}\left[\eta \cdot \left(\frac{\partial u}{\partial y}+\frac{\partial v}{\partial x}\right)\right]+\frac{\partial }{\partial z}\left[\eta \cdot \left(\frac{\partial w}{\partial x}+\frac{\partial u}{\partial z}\right)\right]$$ (7)

$$\rho \cdot \left(u\frac{\partial v}{\partial x}+v\frac{\partial v}{\partial y}+w\frac{\partial v}{\partial z}\right)=-\frac{\partial p}{\partial y}+\frac{\partial }{\partial y}\left[\eta \cdot \left(2\cdot \frac{\partial v}{\partial y}-\frac{2}{3}\cdot \left(\nabla \cdot \vec{v}\right)\right)\right]+\frac{\partial }{\partial z}\left[\eta \cdot \left(\frac{\partial v}{\partial z}+\frac{\partial w}{\partial y}\right)\right]+\frac{\partial }{\partial x}\left[\eta \cdot \left(\frac{\partial u}{\partial y}+\frac{\partial v}{\partial x}\right)\right]$$ (8)

$$\rho \cdot \left(u\frac{\partial w}{\partial x}+v\frac{\partial w}{\partial y}+w\frac{\partial w}{\partial z}\right)=-\frac{\partial p}{\partial z}+\frac{\partial }{\partial z}\left[\eta \cdot \left(2\cdot \frac{\partial w}{\partial z}-\frac{2}{3}\cdot \left(\nabla \cdot \vec{v}\right)\right)\right]+\frac{\partial }{\partial x}\left[\eta \cdot \left(\frac{\partial w}{\partial x}+\frac{\partial u}{\partial z}\right)\right]+\frac{\partial }{\partial y}\left[\eta \cdot \left(\frac{\partial v}{\partial z}+\frac{\partial w}{\partial y}\right)\right]$$ (9)

Pressure inlet and outlet BC are used at the inlet and the outlet, and the pressure values are the same as Eq (4).

Considering the viscous dissipation heating effect in the oil film, the three-dimensional steady-state energy equation is expressed as follows:

$$\text{u}\frac{\partial T}{\partial x}+\text{v}\frac{\partial T}{\partial y}+\text{w}\frac{\partial T}{\partial z}=\frac{\lambda }{\rho C_{p}}\left(\frac{\partial^{2}T}{\partial x^{2}}+\frac{\partial^{2}T}{\partial y^{2}}+\frac{\partial^{2}T}{\partial z^{2}}\right)+\frac{\Phi }{\rho C_{p}}$$ (10)

where Φ represents the dissipation term. The expression can be written as follows:

$$\Phi =2\eta \left[{\left(\frac{\partial \text{u}}{\partial x}\right)}^{2}+{\left(\frac{\partial \text{v}}{\partial y}\right)}^{2}+{\left(\frac{\partial \text{w}}{\partial z}\right)}^{2}+\frac{1}{2}{\left(\frac{\partial \text{u}}{\partial y}+\frac{\partial \text{v}}{\partial x}\right)}^{2}+\frac{1}{2}{\left(\frac{\partial \text{u}}{\partial z}+\frac{\partial \text{w}}{\partial x}\right)}^{2}+\frac{1}{2}{\left(\frac{\partial \text{v}}{\partial z}+\frac{\partial \text{w}}{\partial y}\right)}^{2}\right]$$ (11)

The local viscosity in Eqs (6,7,8 and 10) is estimated by using Eq (2).

The thermal boundary conditions are as follows:

$${T|}_{z=0}=T_{b},\;{T|}_{z=\delta }=T_{f},\;{T|}_{x=0}=T_{\text{inlet}}$$ (12)

#### Thermal equilibrium equations

The thermal balance of viscous dissipation *Q*_*vis*_, conduction *Q*_*b*_, *Q*_*f*_ and convection *Q*_*conv*_ in the oil film can be written as follows:

$$Q_{b}+Q_{vis}+Q_{f}+Q_{conv}=0$$ (13)

where

$$Q_{f}=\int_{x=0}^{x=B}\int_{y=0}^{y=L}-\lambda {\frac{\partial T}{\partial z}|}_{z=\delta }dxdy$$

$$Q_{vis}=\int_{x=0}^{x=B}\int_{y=0}^{y=L}\int_{z=0}^{z=\delta }\Phi dxdydz$$

$$Q_{b}=\int_{x=0}^{x=B}\int_{y=0}^{y=L}-\lambda {\frac{\partial T}{\partial z}|}_{z=0}dxdy$$

$$Q_{conv}=\int_{y=0}^{=L}\int_{z=0}^{z=\delta }\left[{\left(\rho \text{u}C_{p}T\right)}_{x=B}-{\left(\rho \text{u}C_{p}T\right)}_{x=0}\right]dydz$$

Because the load F is not very large and all of the calculations in this paper are within the range of laminar flow, the elastic deformation and turbulence effects are neglected. Furthermore, the presented simulation model includes the influences of reverse flow at the oil inlet and outlet on the THD lubrication performance.

### Numerical Procedure

To calculate the oil film thickness *δ* and the pressure distribution within it, a program is written using MATLAB to solve the Reynolds equation by the finite difference method. The main stages in the numerical procedure are summarized as follows:

1. The minimum oil film thickness *δ*_0_ was assumed, and the viscosity *η* was calculated according to the oil temperature *T*_n_ (in the first calculation, *T*_0_ = *T*_inlet_). Then the two-dimensional pressure distribution in the oil film was determined using the program mentioned above.
2. The integral value of the pressure distribution was calculated and compared with the applied load F. If they were not equal, the value of *δ*_0_ was modified, and step (1) was iterated until the calculation converged (residuals of F < 0.5N).
3. The value of *δ*_0_ was used to determine the domain of the oil film thickness, and the three-dimensional CFD grid was established.
4. The Navier-Stokes equations and energy equations were solved by the Fluent 13.0 solver.
5. The calculated volume-weighted average temperature *T*_n+1_ are compared with the *T*_n_. If the difference between them are larger than 0.1°C, step (1)-(4) were repeated. Usually 4–5 iterations were needed to reach convergence.
6. The velocity and temperature distributions *Q*_*f*_, *Q*_*b*_, *Q*_*vis*_, and *Q*_*conv*_ were calculated.

**Fig 2:**
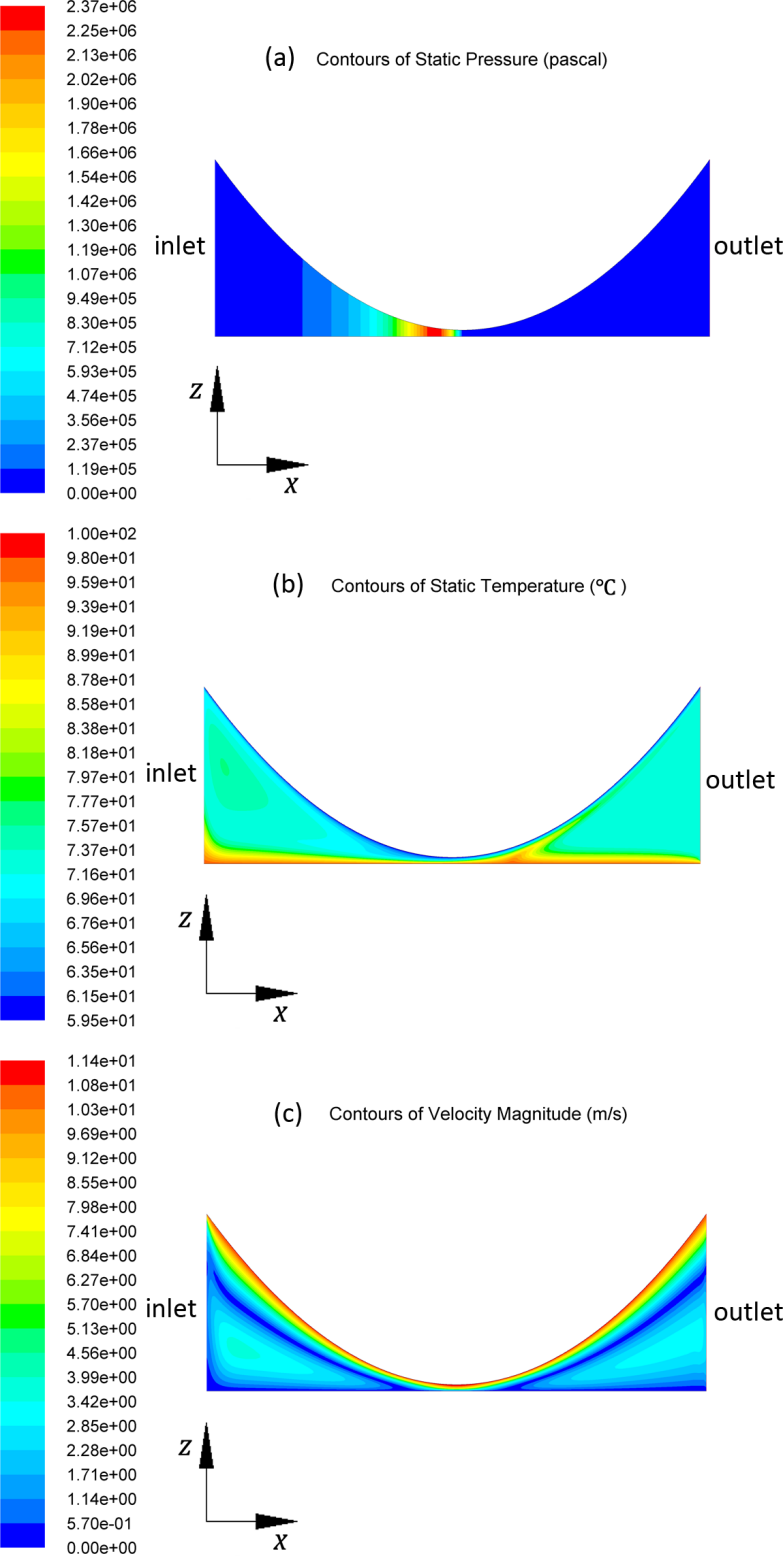
Contours of pressure, temperature and velocity distribution.

For example, the pressure, temperature and velocity distribution at the mid-plane(y = L/2) of oil film, with boundary conditions of U = 11.40 m/s, *T*_inlet_ = 70°C, F = 300 N, and *T*_*b*_ = 100°C, are shown in Fig 2. The scale in the z axis direction(thickness direction) was enlarged 20 times for display. In the contours of pressure, pressure in the diverging area is set to 0 by applying the Half Sommerfeld Boundary Condition. In the contours of temperature, the convective effect can be seen at left side of the minimum oil film area.

### Grid Independence Analysis

**Fig 3:**
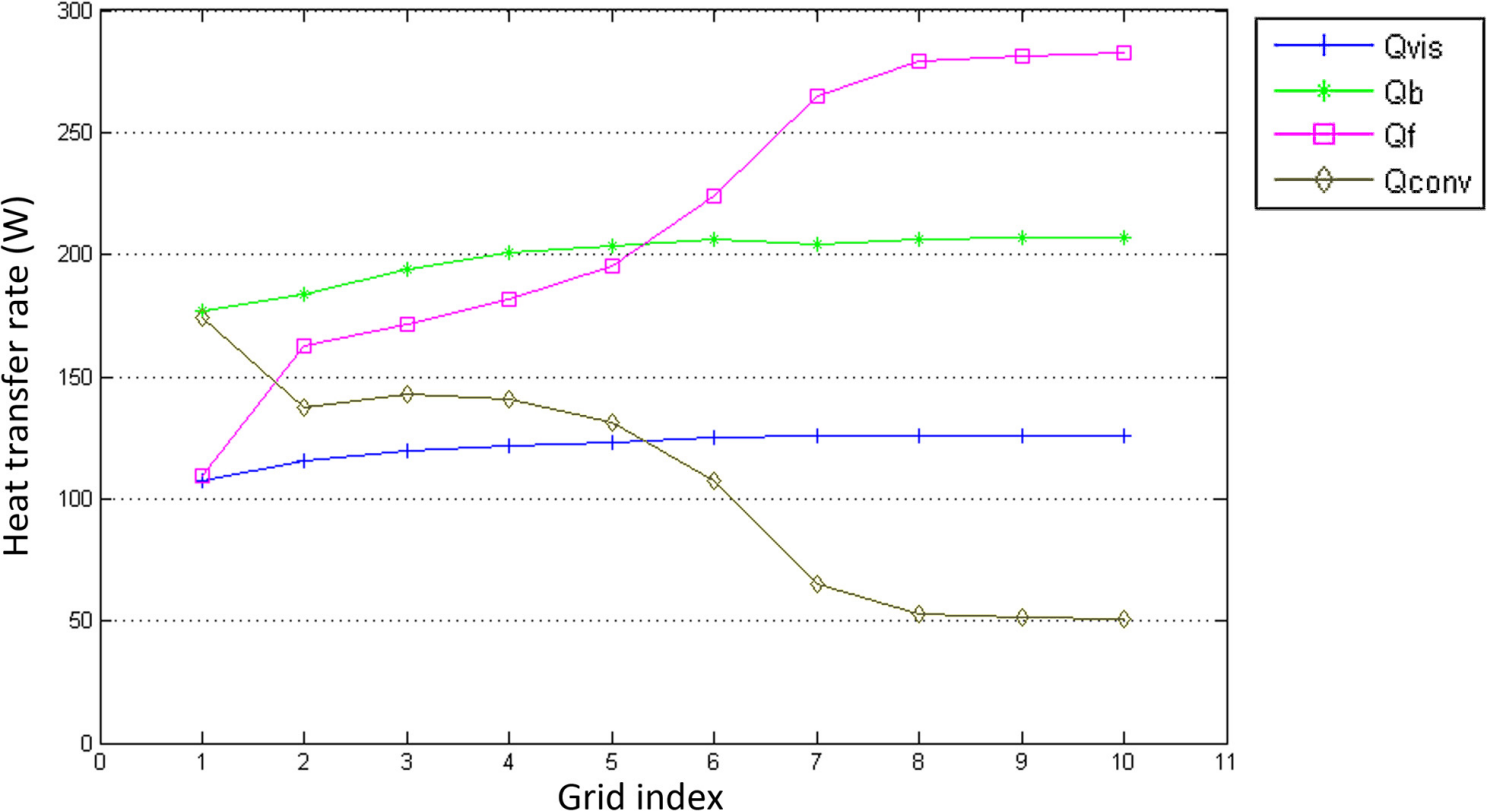
Results of the grid independence calculation.

In this paper, 10 structured grid schemes have been used to reach grid independency, and the details of these grids are shown in Table 3. With boundary conditions of U = 11.40 m/s, *T*_inlet_ = 70°C, F = 300 N, and *T*_*b*_ = 100°C, the calculated *δ*_0_ was 12.43 μm. The Laminar viscous model, the SIMPLEC algorithm, the no slip wall shear condition were utilized. The results of the calculation in Fig 3 shows that the results of grids #8, #9 and #10 are very similar, thus further refinement of the grid would barely influence the simulation results. Therefore, the #8 grid discretization scheme was used in further studies in this paper.

**Table 3:** Grid resolutions.

Grid index	Grid number(X×Y×Z)
#1	200×400×2
#2	200×400×3
#3	200×400×4
#4	300×500×5
#5	400×600×6
#6	800×2000×10
#7	800×2000×20
#8	1600×4000×40
#9	1600×4000×60
#10	2000×4000×80

### Experimental apparatus

**Fig 4:**
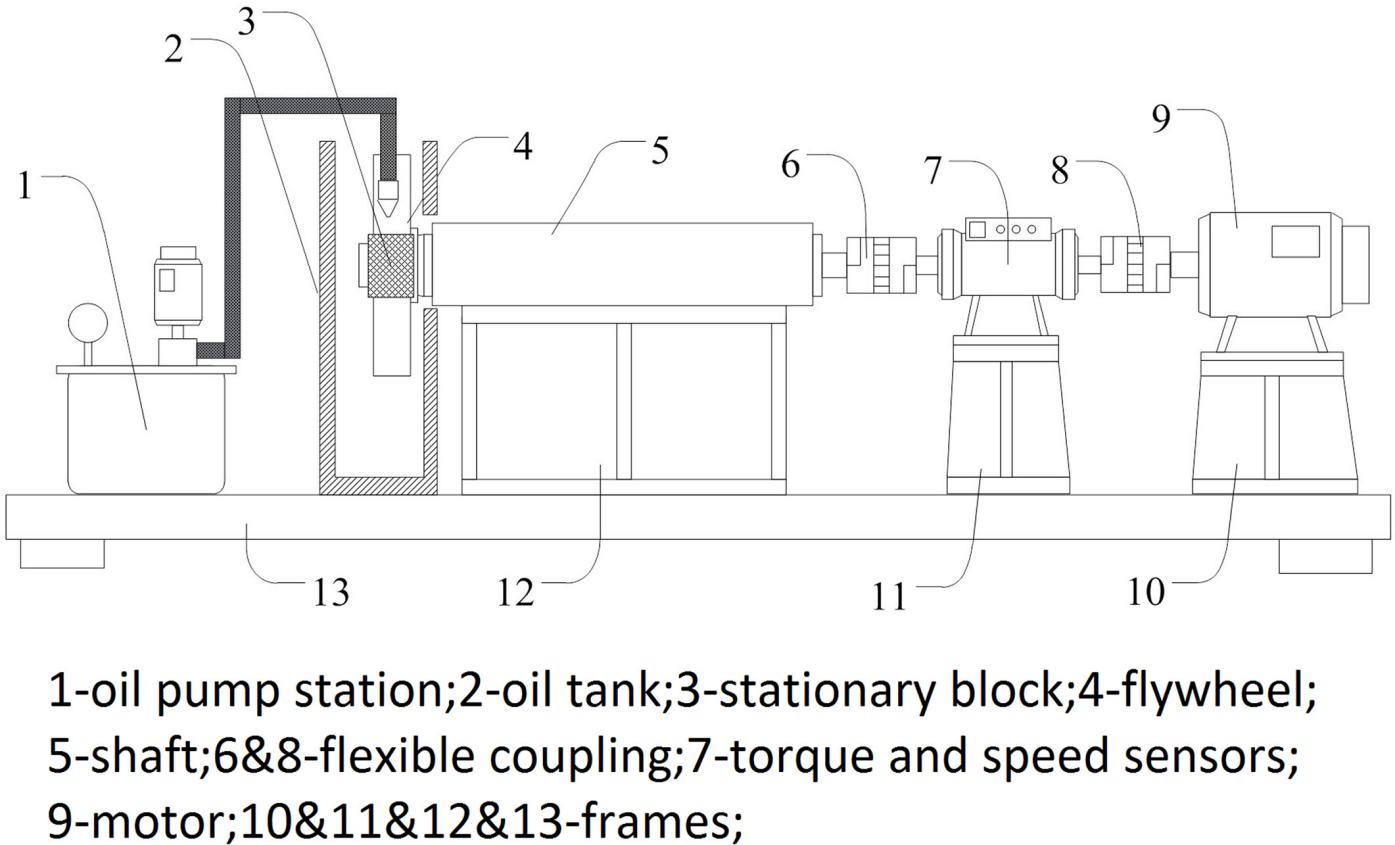
Schematic diagram of the apparatus.

**Fig 5:**
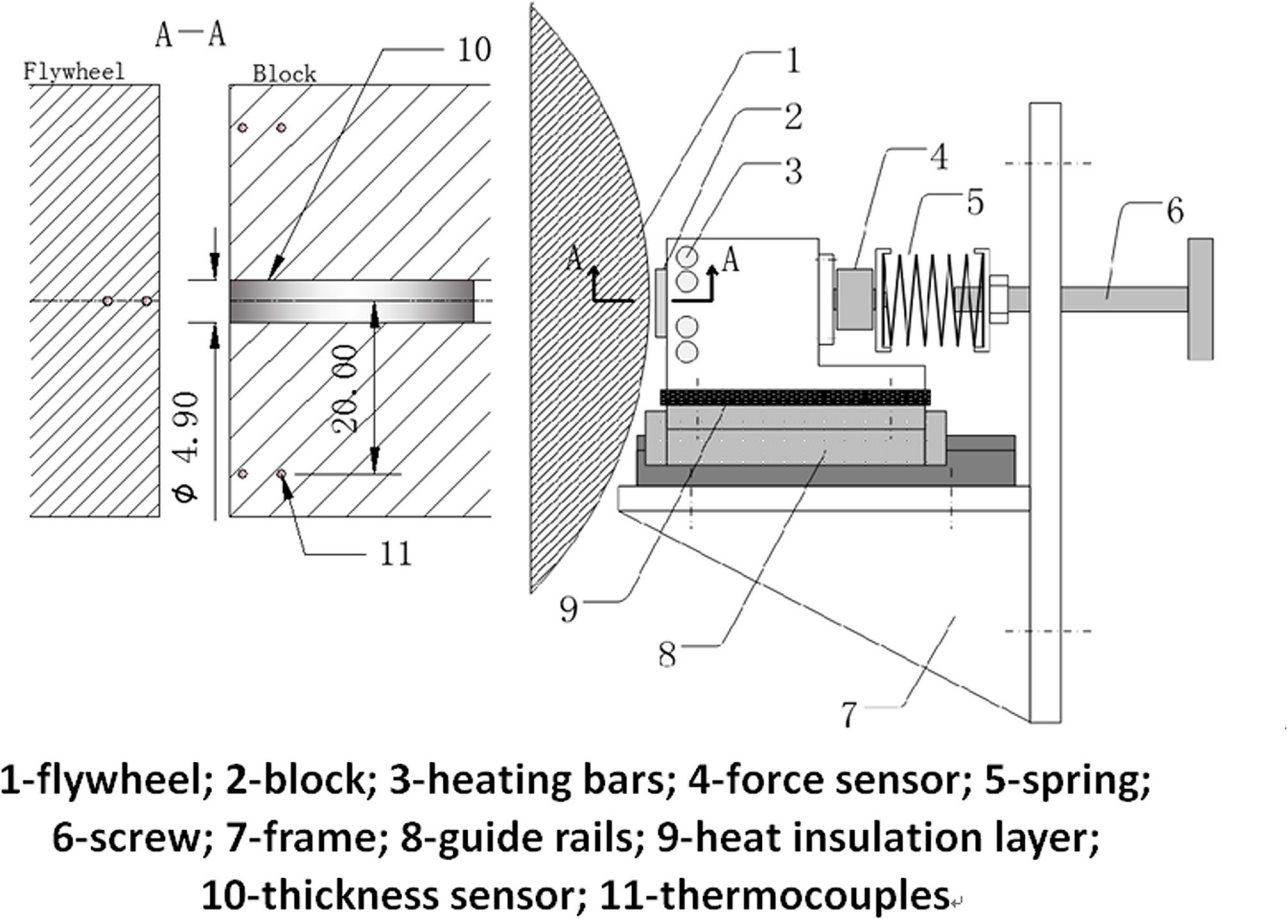
Schematic diagram of the sensors.

An experimental apparatus was developed to validate the credibility of the calculated results. The schematic of the test system are shown in Fig 4 and Fig 5. The flywheel was used to imitate the cylindrical surface’s rotation. Four electric heating bars were buried in the block to adjust *T*_*b*_. A spring-rail mechanism was designed to restrict the block to slide only in the *z* axis direction and to generate the applied load F at the same time. The force sensor and the capacitance type sensor (Fig 5) were used to measure the applied load F and the oil film thickness *δ*_0_, respectively, and the parameters of these sensors are shown in Table 4.

**Table 4:** Parameters of the force sensor and the thickness sensor.

Parameters	Force sensor	capacitance type thickness sensor
Range of measurement	±323 N	±30 μm
Resolution	-	0.01 μm
Output signal	±20 mV	±5 V
Nonlinearity	±0.5%	±0.5%
Accuracy	±1%	±1%
Frequency response	-	7500 Hz

The surface temperatures *T*_*b*_ and *T*_*f*_ were measured by k-type thermocouples. The first-layer thermocouples were placed 1.5 mm from the metal surfaces, and the second-layer thermocouples were 6 mm from those surfaces. The temperature signals on flywheel side were saved by an storage measurement system and uploaded into the computer after experiments. The storage measurement system can ensure the time-synchronization between the temperature signals of both sides. Then, *T*_*b*_ and *T*_*f*_ were acquired by an interpolation method. The shaft torques M and M_0_ were also measured. Because the measurements were under steady-state conditions, the motor power consumption was considered to balance the viscous dissipation which could be written as follows:

$$f=\frac{1}{R_{f}}(M-M_{0})$$ (14)

$$Q_{vis}=f\cdot U$$ (15)

15W40 oil was used as the test lubricant in this paper. The oil could be heated within the pump station, and the oil temperature was controlled at a stable value (the maximum temperature fluctuation ≤ 0.94°C).

## Results and Discussion

### Comparison between experimental data and numerical results

**Fig 6:**
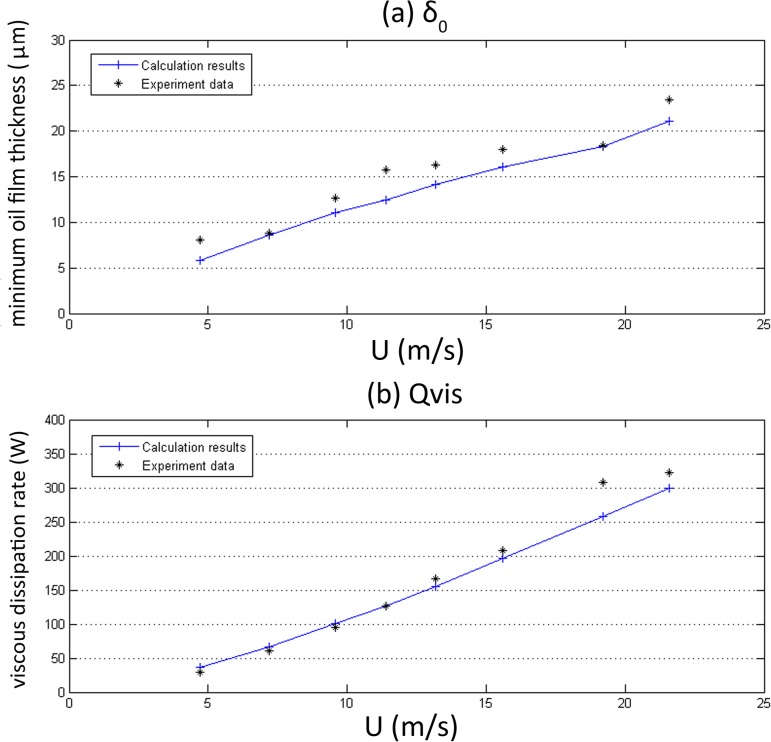
Comparison between experimental data and numerical results.

The experimental conditions, which were also applied in the calculation, are shown in Table 5. Fig 6 shows the comparison between the experimental data and the numerical results for *δ*_0_ and *Q*_*vis*_. The trends and the values agree well. The difference between the experimental data and the numerical results is less than ±13%, except for #1, #4 and #7, which might be caused by vibration of the working flywheel at specific speeds. The experiment data verified the credibility of the simulation model, thus this simulation model was adopted in the following studies.

**Table 5:** Cases for the influence of the speed.

Case	#1	#2	#3	#4	#5	#6	#7	#8
Flywheel speed (r/min)	300	458	611	726	840	993	1222	1375
F (N)	300	300	300	300	300	300	300	300
*T*_inlet_ (°C)	70	70	70	70	70	70	70	70
*T*_*b*_ (°C)	100	100	100	100	100	100	100	100
*T*_*f*_ (°C)	56.83	58.52	58.75	59.48	59.6	59.84	60.64	62.23
U (m/s)	4.71	7.19	9.60	11.40	13.19	15.60	19.19	21.60
Reynolds number	17.7	28.0	38.3	48.1	56.4	69.3	92.0	111.8

### Effect of the Reynolds number

The Reynolds number is defined by the expression[24]:

$$Re=\frac{\text{U}\rho \bar{\delta }}{\eta }$$ (16)

Where

$$\bar{\delta }=\frac{\int_{\text{x}=0}^{\text{x}=\text{B}}\;\delta \;\text{dx}}{\text{B}}$$ (17)

**Fig 7:**
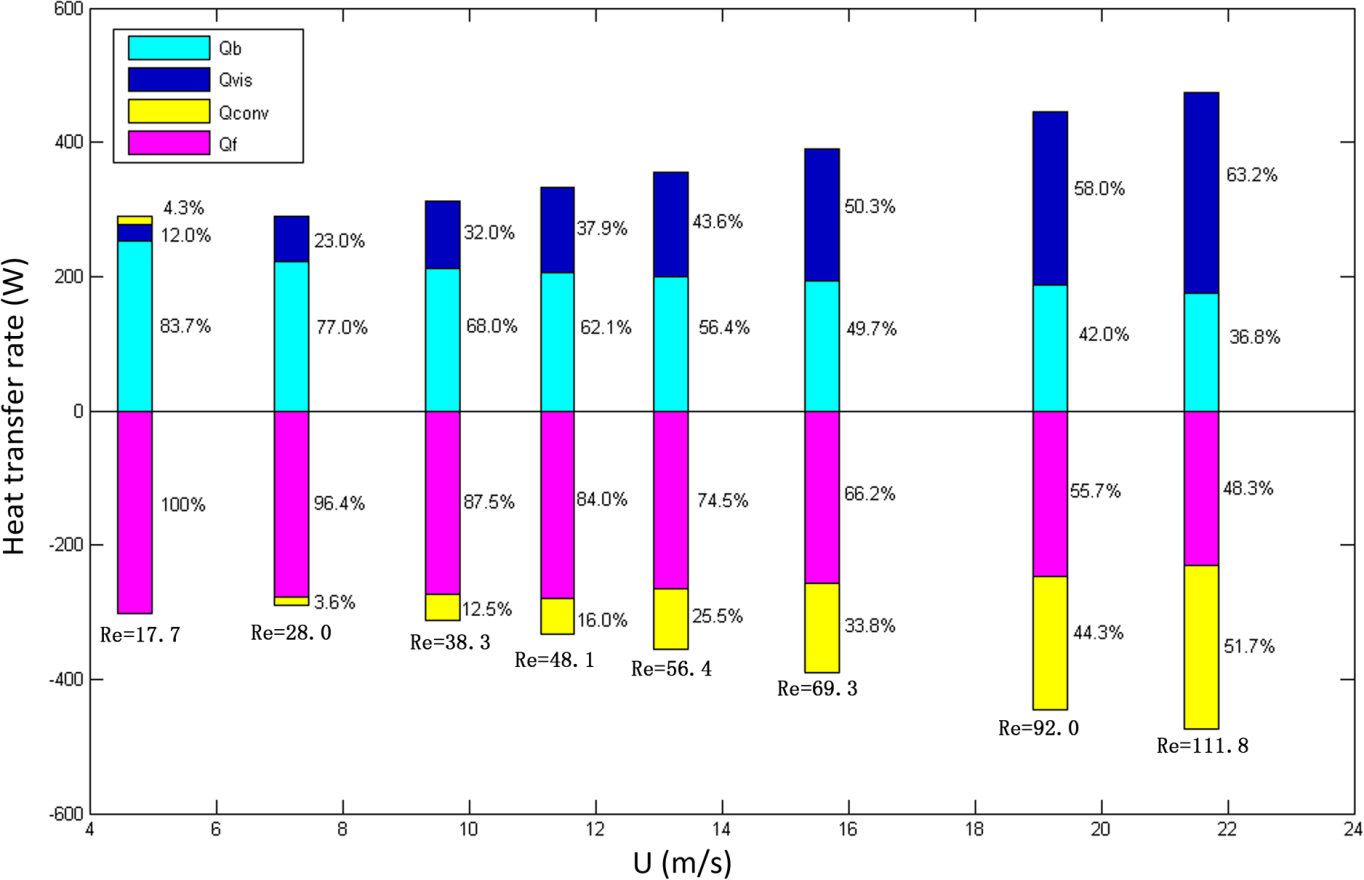
The variations of *Q*_*b*_, *Q*_*f*_, *Q*_*conv*_ and *Q*_*vis*_ with different cylindrical surface linear speed U.

The cases shown in Table 5 were used to study the effect of the Reynolds number on the thermal effects of line contact THD lubrication. *T*_*f*_ data in Tables 5, 6 and 7 were measured and then taken as boundary conditions for calculations. Fig 7 shows the calculated results for the conduction rates *Q*_*b*_, *Q*_*f*_, the convection rate *Q*_*conv*_ and the viscous dissipation rate *Q*_*vis*_. In Fig 7, positive values represent heat flowing into the computational domain or generated within it, whereas negative values represent heat dissipated within the computational domain or flowing out of it.

**Table 6:** Cases for the influence of the temperatures.

Case	#1	#2	#3	#4	#5	#6	#7	#8	#9	#10	#11	#12
U (m/s)	19.19											
F (N)	300											
*T*_*b*_ (°C)	100				80				60			
*T*_inlet_ (°C)	60	70	80	90	60	70	80	90	60	70	80	90
*T*_*f*_ (°C)	60.72	60.64	56.15	58.27	55.92	53.17	56.15	57.15	49.77	54.95	56.49	55.12

**Table 7:** Cases for the influence of the load capability.

Boundary conditions	U (m/s)	F (N)	F*	*T*_*b*_ (°C)	*T*_inlet_ (°C)	*T*_*f*_ (°C)
#1	4.71	220	0.199	100	70	55.36
#2		260	0.230			56.52
#3		300	0.265			56.83
#4		340	0.294			57.82
#5		380	0.326			58.46
#6	19.19	220	0.073			59.54
#7		260	0.082			60.41
#8		300	0.092			60.64
#9		340	0.101			61.39
#10		380	0.111			61.82
#11	21.60	220	0.070			61.27
#12		260	0.079			61.75
#13		300	0.088			62.23
#14		340	0.097			62.59
#15		380	0.105			62.89

In Fig 7, *Q*_*b*_ and *Q*_*f*_ decrease when the Re increases, whereas *Q*_*conv*_ and *Q*_*vis*_ increase remarkably. Under low Re conditions, most of the heat is transferred by conduction. For example, the proportions of *Q*_*b*_ and *Q*_*f*_ in the thermal effects are over 80% when Re = 17.7, and therefore conduction plays a primary role in heat transfer under low Re conditions, meanwhile *Q*_*conv*_ plays a very small role (4.3%) in bring heat into the oil film. However, when the Re is increased to 111.8, the contribution of the convection heat transfer rate *Q*_*conv*_ to the heat dissipation is more than 50%. The proportion of the viscous heating rate *Q*_*vis*_ increases dramatically with the Re.

To explain these results, the variation of the minimum oil film thickness *δ*_0_ is shown in Fig 6. The value of *δ*_0_ increases with the linear speed U, which agrees with the results of Dowson et al. [8]. Because *δ*_0_ increases, the temperature gradient in the *z* axis direction decreases near both sides of the metal surfaces leading to a decrease of *Q*_*b*_ and *Q*_*f*_. Conversely, the oil shear rate between these two metal surfaces increases according to Eq 10, which results in a significant increase of the viscous heating rate *Q*_*vis*_. Because the oil flow rate also increases with the linear speed U, the convection heat transfer rate *Q*_*conv*_ increases from 3.6% to 51.7%, which means convection becomes significant in heat dissipation.

According to the discussion above, we can conclude that convection and viscous heating play important roles in the thermal effects of line contact THD lubrication, and the influence is greater for higher relative speed of the metal surfaces. The influence cannot be neglected during line contact THD lubrication analysis; otherwise, the calculation will include large errors and cannot reflect reality, especially under high Re conditions.

### Effect of temperature

This section discusses the influences of the oil inlet temperature *T*_inlet_ and the surface temperatures *T*_*b*_ and *T*_*f*_ on the line contact THD lubrication as studied using the cases shown in Table 6. The speed U and the applied load F remained constant, and *T*_*b*_ was set to 60, 80 and 100°C, respectively.

**Fig 8:**
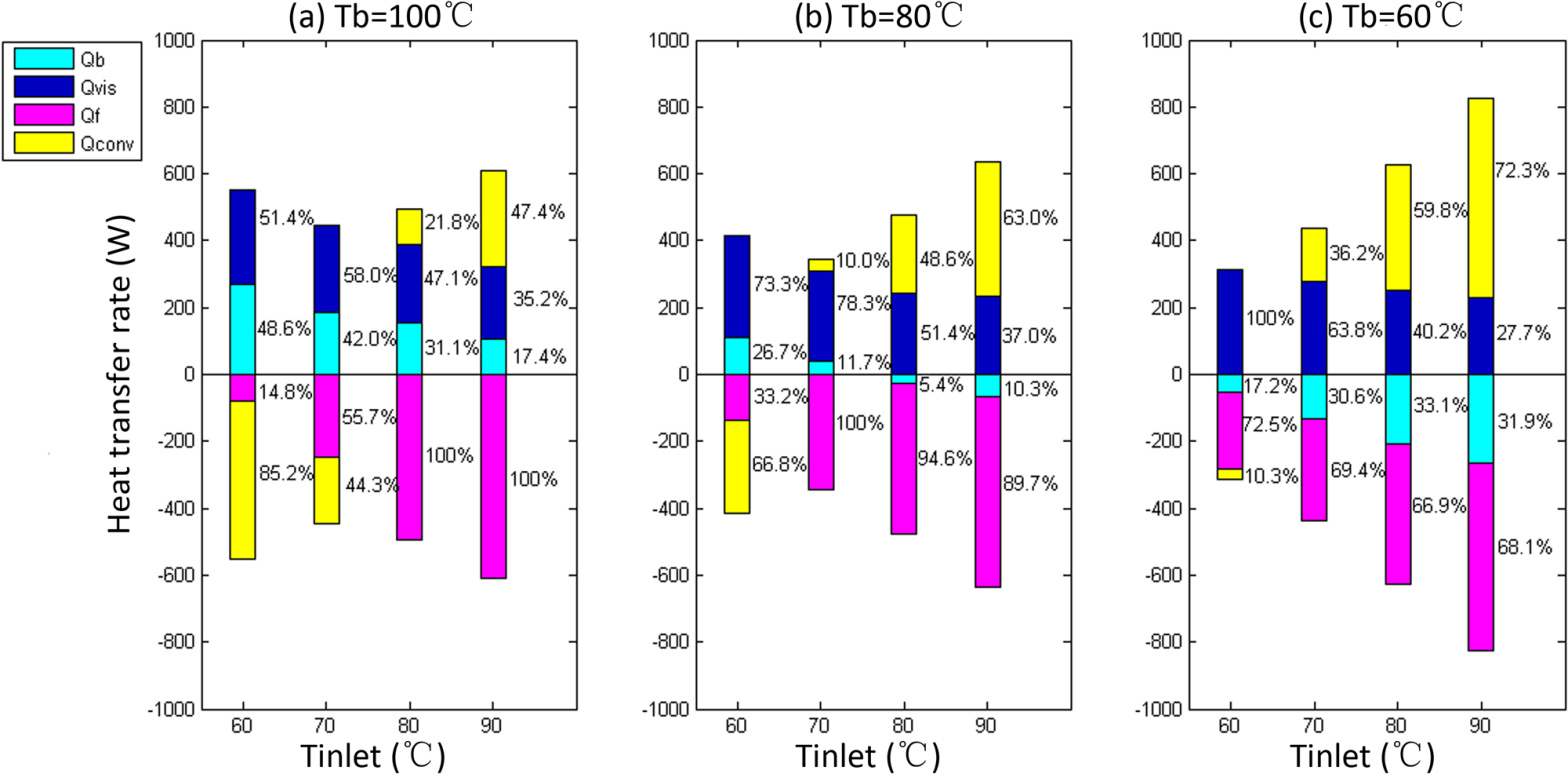
The variations of *Q*_*b*_, *Q*_*f*_, *Q*_*conv*_ and *Q*_*vis*_ with different oil inlet temperature *T*_inlet_.

**Fig 9:**
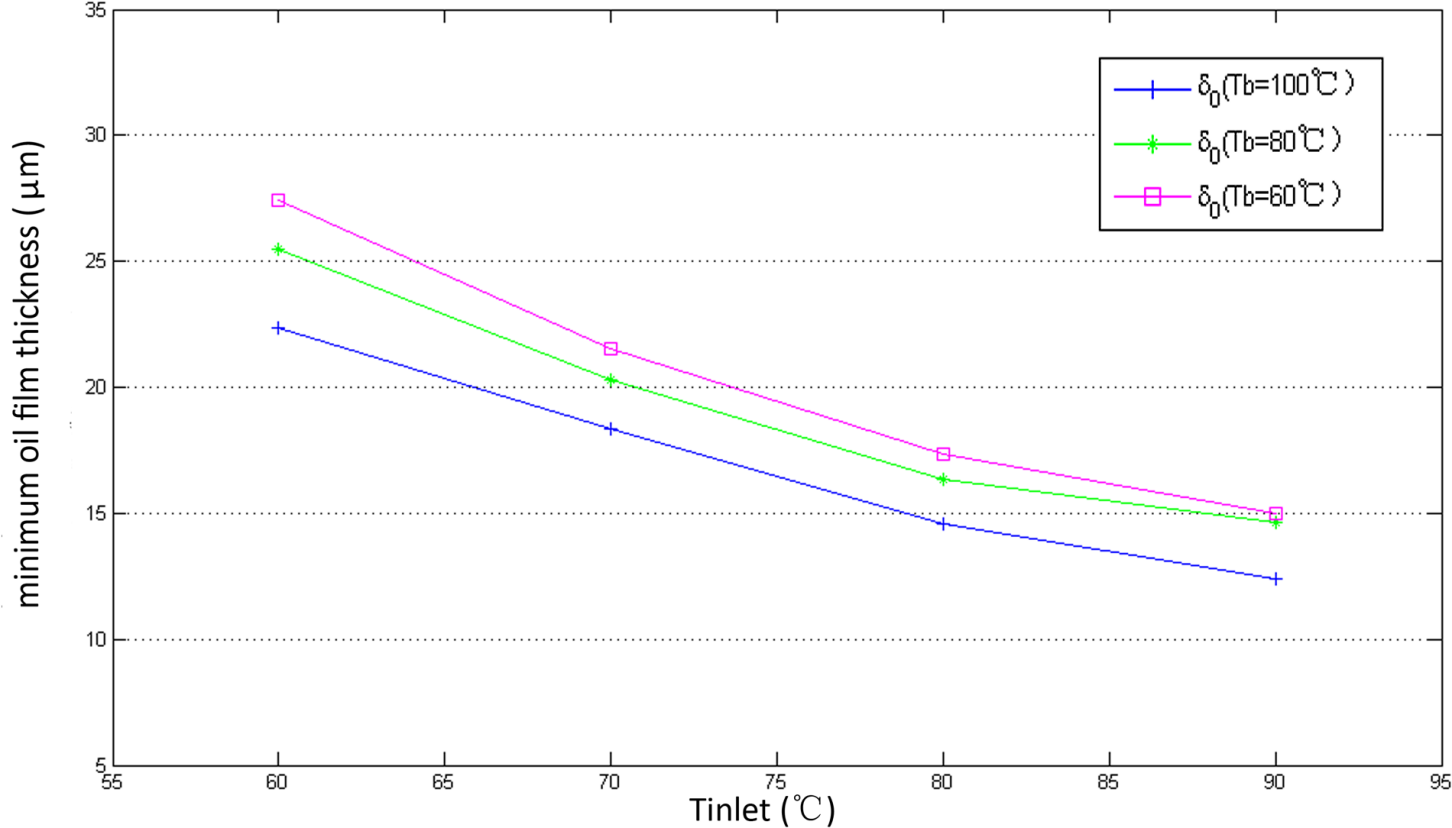
The variation of *δ*_0_ with different oil inlet temperature *T*_inlet_.

The results are shown in Fig 8. With increasing *T*_inlet_, the effect of *Q*_*conv*_ in the thermal effects changes from dissipating heat to bringing heat into the oil film. The point of conversion depends on the difference of *T*_inlet_ and *T*_*b*_: If *T*_inlet_ >> *T*_*b*_, *Q*_*conv*_ brings heat; If *T*_inlet_ << *T*_*b*_, *Q*_*conv*_ dissipates heat; however, if the difference between *T*_inlet_ and *T*_*b*_ is small, it is difficult to deduce the direction (in or out) of heat flow without careful calculations. The calculation results for *Q*_*b*_ reflect the same situation. With increasing *T*_inlet_, the value of *Q*_*vis*_ decreases slightly, but the proportion of *Q*_*vis*_ in the thermal effects changes dramatically. These results can be explained as follows: According to the research of Harigaya et al. [19], the minimum oil film thickness *δ*_0_ decreases with increasing *T*_inlet_ due to decreasing oil viscosity as shown in Fig 9. However, the decrease of *δ*_0_ results in an increase of the shear rate in the oil film. Combining these two factors, the total amount of *Q*_*vis*_ fluctuates slightly. Meanwhile, other effects fluctuate rapidly with variation of *δ*_0_, resulting in the fluctuation of the proportion of *Q*_*vis*_ with temperature.

From Fig 8 it can be concluded that *Q*_*b*_, *Q*_*f*_, *Q*_*conv*_ and *Q*_*vis*_ are coupled. The quantity and the direction of heat flux near the metal surfaces are determined not only by the temperature difference between the metal surfaces but also by the inlet temperature. Thus, *Q*_*b*_, *Q*_*f*_ and the flow directions should be calculated for specific cases, and the proportions of *Q*_*b*_, *Q*_*f*_, *Q*_*conv*_ and *Q*_*vis*_ vary greatly with the boundary conditions. In some cases in the literature [25–27], the convection heat transfer effect was neglected and replaced with a one-dimensional thermal resistance model in THD lubrication analysis, and the quantity and the direction of the heat flux were determined only by the difference between the temperatures of the metal surfaces. This type of simplified method may not represent the real physical processes, and the calculation results need to be further modified.

### Effect of applied load

The dimensionless load is defined by the expression[28]:

$${\text{F}}^{*}=\frac{{\bar{\delta }}^{2}F}{6\eta UB^{2}L}$$ (18)

The cases for analysis of the influence of applied load are shown in Table 7, where the block surface temperature *T*_*b*_ and oil inlet temperature *T*_inlet_ remained constant, and U was set separately to 4.71, 19.20, and 21.60 m/s.

**Fig 10:**
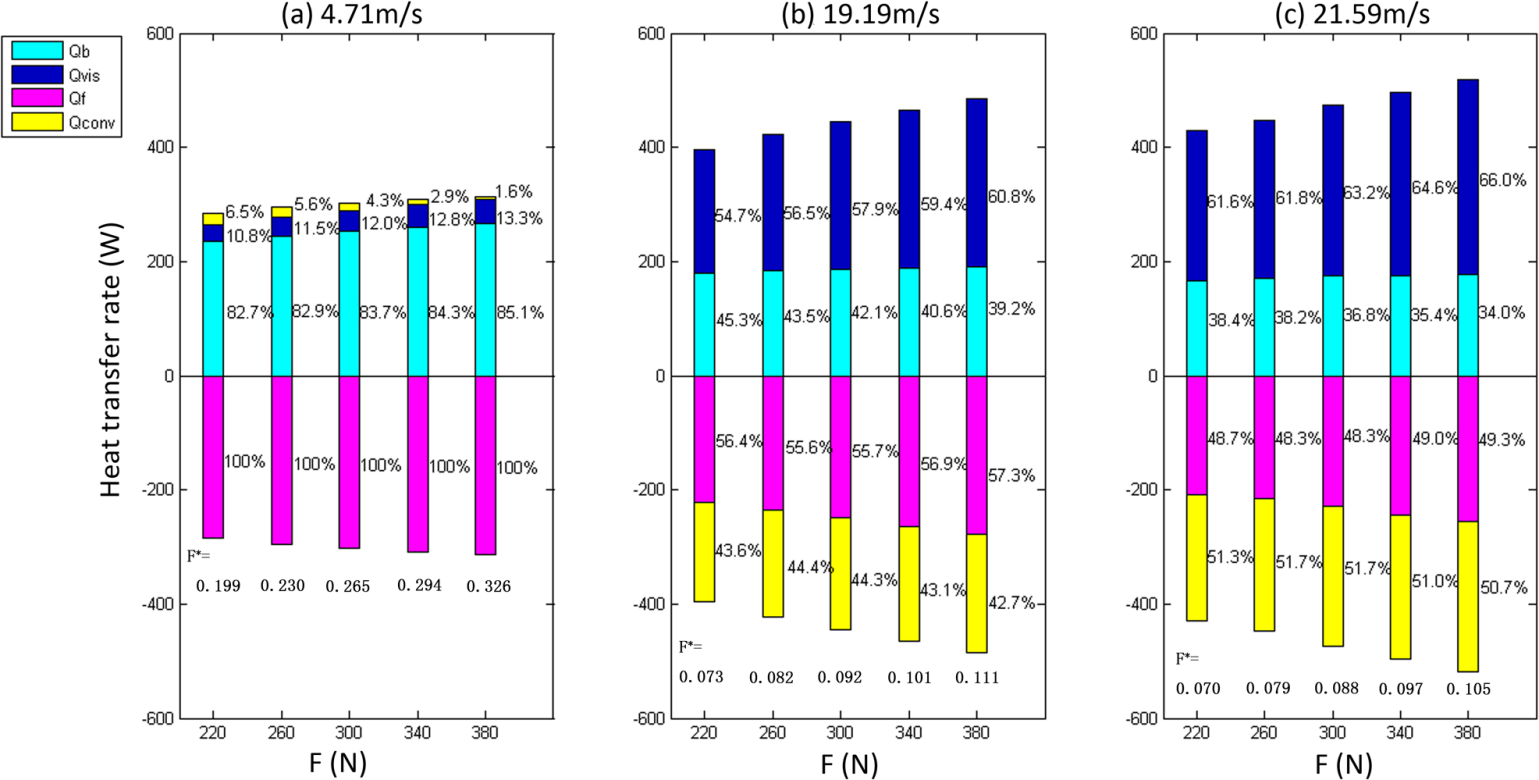
The variations of *Q*_*b*_, *Q*_*f*_, *Q*_*conv*_ and *Q*_*vis*_ with different load force F.

**Fig 11:**
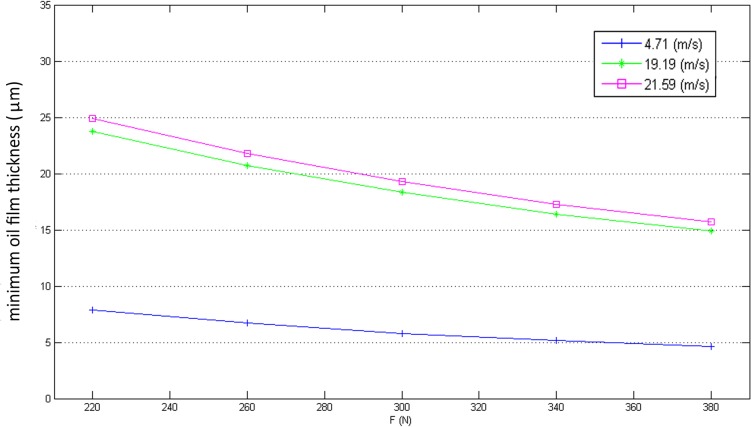
The variations of *δ*_0_ with different load force F.

Fig 10 shows the calculated results of *Q*_*b*_, *Q*_*f*_, *Q*_*conv*_ and *Q*_*vis*_ for various values of applied load F. The results show that the values of *Q*_*b*_ and *Q*_*f*_ increase, and their proportions of the thermal effects change slightly with increasing applied load F. For low linear speed U (4.71 m/s), *Q*_*vis*_ does not vary obviously with increasing F, but at high linear speed, *Q*_*vis*_ increases far more obviously with applied load F. These results can be explained through the calculated variation of oil film thickness, which is shown in Fig 11 and is consistent with the previous studies [29]. The minimum oil film thickness *δ*_0_ decreases when the applied load F increases regardless of the cylindrical surface speed U, but the degree of variation of *δ*_0_ under high speed conditions is much larger than under low speed conditions. This behavior of *δ*_0_ leads to two consequences: 1) a higher temperature gradient near the metal surfaces and 2) a higher shear rate in the oil film, which causes *Q*_*b*_, *Q*_*f*_ and *Q*_*vis*_ to all increase. However, regarding the proportions, *Q*_*vis*_ changes more than the others, so the proportions of *Q*_*b*_ and *Q*_*f*_ decrease slightly. The value and proportion of *Q*_*conv*_ both increase, but not obviously, with the applied load F.

The calculation results for different linear speed U conditions show the same tendencies, with slight differences in the magnitudes and proportions. The results indicate that the proportions of *Q*_*b*_, *Q*_*f*_, *Q*_*conv*_ and *Q*_*vis*_ in the thermal effects remain almost unchanged with variation of the applied load F, whereas the cylindrical surface speed U has a greater effect on these proportions. From the view of dimensionless load, the proportion of conduction decreases while the proportions of convection and viscous dissipation increases with the increasing of F*.

## Conclusion

To address the disagreements on the proportions of conduction, convection and viscous dissipation effects at various friction conditions in former studies, a simplified steady-state THD analysis model of line contact lubrication was developed to analyze the influences of Reynolds number, applied load, oil inlet temperature and temperatures of the metal surfaces.The calculation results agree well with the experimental data. The following conclusions can be drawn:

The convective heat transfer rate and viscous dissipation heating rate was found increase with the Re. The proportion of convection in the thermal effects of line contact lubrication could be more than 50% under high Re conditions. It proves that the effect of convection on high Re THD lubrication analysis is significant and should not be neglected.

In some situations wherein the temperatures of the inlet oil and the friction pair surfaces are quite close, the quantity and direction of conductive and convective heat flow should be determined through calculation with specific boundary conditions, instead of being estimated from the difference of the friction pair surface temperatures. The one-dimensional thermal resistance model can not reflect the real heat transfer process.

The applied load has little impacts on thermal effect. The viscous heating rate increases with applied load slightly. The conductive and convective heat transfer rate remains almost constant with varying applied load. From the view of dimensionless load, the proportion of conduction decreases while the proportions of convection and viscous dissipation increases with the increasing of F*.

In further studies, the influence of the temporal variation of the relative speed and roughness of surfaces would be taken into account. Additionally, turbulence and cavitation effects could be included in the coming studies.
